# Hysteria in Connexion with the Belfast Revival
*The Work and the Counterwork, or the Religious Revival in Belfast, with an Explanation of the Physical Phenomena. By Edward A. Stopford, Archdeacon of Meath. Third Edition, 8vo; pp. 104. Dublin: Hodges, Smith, & Co., 104, Grafton Street.


**Published:** 1859-10-01

**Authors:** 


### Art. IX.—HYSTERIA IN CONNEXION WITH THE BELFAST REVIVAL.*

The revival of religion that is still progressing in the North of Ireland has been attended by many instances of the nervous disorders which appear to be almost inseparable from similar movements; and we find, in the pamphlet before us, a praiseworthy endeavour to explain, to the clergy and parents, the line of demarcation between the beneficial effects of awakened religious feeling and the rhapsodies of hysterical or cataleptic ecstasy.

Our readers will not require to be told that, in nearly all the "revivals" upon record, certain phenomena indicative of diseases of the nervous system have been manifested by many individuals. On such occasions the widely-spread rumours of miraculous conversions and beatific visions exercise a magnetic effect upon the weak and credulous, and attract to the camp meetings, the out-door preachings, or other centres of excitement, a large proportion of persons who are prepared to surrender themselves to any morbid impulse. As the business of such meetings proceeds, the vehement language from the pulpit, the exclamations or lamentations of some among the worshippers, and the notoriety accorded to conspicuous "cases," combine to produce all the most efficient excitants of hysteria; and are too frequently followed by some of its Protean forms, and some of its direful consequences. Most commonly, as, for instance, among the early Methodists, and in the revival which took place in America at the beginning of the present century, the instances of hysteria have been hailed as special manifestations of Divine grace; and the slaves of the most self-seeking of all diseases have been regarded as the elect saints of God. From the vantage ground of such a position they have been able to repel the scrutiny of scientific observers, to avoid any close inspection of the morbid phenomena really occurring, and to perfect any scheme of imposture that might tend to inflame the fervid ignorance, or to refresh the waning zeal, of the enthusiasts by whom they were surrounded. The medical records of these cases are therefore extremely meagre; and physicians, aware of the profligacy and insanity to which any large aggregate of hysteria must give rise, have probably placed themselves too much in a position of antagonism to great religious movements as a whole; so as to lose, in some measure, the confidence of the communities that such movements sway; and to forfeit the power of indicating and controlling mere physical disease, which it should be the province of their profession to exert.

Such was lately the state of affairs at Belfast. Catalepsy, and cataleptic ecstasy, were matters of frequent occurrence at certain places of worship, and were daily on the increase. Mill-girls were praying to

* The Work and the Counterwork, or the Religious Revival in Belfast, with an Explanation of the Physical Phenomena. By Edward A. Stopford, Archdeacon of Meath. Third Edition, 8vo; pp. 104. Dublin: Hodges, Smith, & Co., 104, Grafton Street.

be "struck." The best "cases" were obtaining a comfortable maintenance by relating their visions to daily levees of admiring auditors. The clergy, of all denominations, were wholly unprepared for the emergency, were ignorant, speaking generally, of what it was they witnessed, and do not appear to have imagined that medical men could have any special knowledge of the causes in operation, or any special duties with regard to the effects produced. Indeed, in the words of a correspondent, all the pious ladies of Belfast would have combined to destroy the practice of a doctor who had expressed a doubt of the Divine origin of the prevailing visitations.

At this conjuncture, Archdeacon Stopford had the discernment to perceive the nature of the physical effects produced by the popular excitement, and the courage to call these effects by their right name. His pamphlet is not less remarkable for its cordial recognition of the devotional movement, than for its indignant denunciation of the attendant hysteria; and is therefore calculated to eliminate the evil from the good, or, as the Archdeacon phrases it, the tares from the wheat, without alarming the prejudices, or alienating the sympathies, of the most ardent Presbyterian or Methodist. He traces some of the attacks of hysteria clearly enough to the abuse of certain pulpit arts, to the reiteration of hell, hell, hell—the accumulation of a depressing emotion, for which no outlet was provided in active thought or practical duty; and others to the kindred but more gradual operation of the mental atmosphere of the locality; and he describes the nature and tendencies of the disease with sufficient clearness to disabuse any candid mind of the supposition that it can ever, under any circumstances, be used as an instrument of good. What is still more important, he points out the manner in which the clergy may so control their congregations as to prevent such outbreaks for the future. Upon these and other parts of the subject, our space does not allow of quotations at sufficient length to do justice to the author; and we must be content most cordially to recommend the pamphlet itself to the attentive perusal of our readers. Among other benefits likely to result from its timely publication, we hope that facilities will be afforded for the medical examination of any cases of catalepsy that may still occur; and that the present revival may be so studied, in its physical aspects, as to throw fresh light upon the most obscure portion of nervous pathology.

It is only necessary to add, that Archdeacon Stopford's pamphlet has now gone through three editions; and that, in the last of these, some trifling inaccuracies on points of physiological detail have been either corrected or removed.

